# Prominent entrapment of respiratory epithelium in primary and metastatic intrapulmonary non-epithelial neoplasms: a frequent morphological pattern closely mimicking adenofibroma and other biphasic pulmonary lesions

**DOI:** 10.1007/s00428-020-02796-7

**Published:** 2020-03-19

**Authors:** Ramona Erber, Florian Haller, Arndt Hartmann, Abbas Agaimy

**Affiliations:** Institute of Pathology, Friedrich-Alexander University Erlangen-Nürnberg (FAU), University Hospital, Krankenhausstrasse 8-10, 91054 Erlangen, Germany

**Keywords:** Sarcoma, Lung metastasis, Adenofibroma, Fibroadenoma, Lung, Nephroblastoma, Osteosarcoma, Germ cell tumor, Adenomyoepithelioma, Hamartoma

## Abstract

As one of the most common target organs for hematogenous spread from diverse cancers, biopsy interpretation of lung tumors is complicated by the challenging question of *primary* versus *metastatic* and by frequent entrapment of native respiratory glands. Nevertheless, the literature dealing with this issue is surprisingly sparse and no single study has been devoted to this topic. We reviewed 47 surgical lung specimens of non-epithelial neoplasms (38 metastases, mainly from sarcomas and 9 primary lesions) for frequency and pattern of intralesional epithelial entrapment. Respiratory epithelium entrapment was noted in 23/47 (49%) cases (diffuse in 15 and peripheral in 8). Entrapped glands frequently showed prominent regenerative and reactive changes mimicking neoplastic glands. Based on cellularity of the mesenchymal component and the extent, distribution and shape of entrapped respiratory glands, four morphological patterns were recognized: paucicellular sclerosing low-grade neoplasms containing leaflet-like glands indistinguishable from adenofibroma and fibroepithelial hamartomas (*n* = 11), and biphasic cellular lesions mimicking adenomyoepithelioma (*n* = 1), biphasic synovial sarcoma (*n* = 2), and pleuropulmonary blastoma (*n* = 1). Only a single genuine pulmonary adenofibroma was identified. This study highlights frequent respiratory epithelium entrapment in diverse non-epithelial lung tumors, both primary and metastatic. Recognition of this finding and use of adjunct IHC combined with clinical history should help to avoid misinterpretation as primary pulmonary biphasic neoplasm or as harmless adenofibroma. The vast majority of morphologically defined lung adenofibromas represent adenofibroma-like variants of histogenetically diverse entities so that a diagnosis of adenofibroma should be rendered only very restrictively and then as a diagnosis by exclusion.

## Introduction

The lungs represent major target organs for metastatic deposits from malignant neoplasms of diverse histogenetic origin from different anatomic sites. Sarcomas in particular are known to have significant predilection for hematogenous spread to the lungs. Accordingly, thoracic imaging represents an integral part of routine staging investigations for newly diagnosed malignant neoplasms and on follow-up for patients with a history of malignancy. As a consequence of increasing use of high-resolution imaging and increasing frequency of thoracoscopic surgery, incidental pulmonary nodules are being increasingly discovered and excised. Familiarity with the diverse patterns of primary and metastatic intrapulmonary non-epithelial neoplasms is mandatory for distinguishing benign from malignant and primary from metastatic diseases.

A subset of incidentally discovered pulmonary nodules, whether solitary or multiple, represent hamartomatous lesions or benign mesenchymal neoplasms, chondroid hamartomas being the most frequent. Pulmonary adenofibroma (PAF) is an exceptionally rare benign fibroepithelial lesion that closely recapitulates Müllerian adenofibroma of the genital tract [1] or fibroadenoma of the breast [2]. Since its first description by Scarff and Gowar in 1944 [3] and delineation as distinctive entity by Suster and Moran in 1993 [1], only a few cases of genuine PAFs have been described [4, 5].

Although entrapment of native pulmonary epithelium by metastatic neoplasms in the lung is well known, in our experience this phenomenon and the pitfalls related to it have not received sufficient attention in the surgical pathology literature, possibly explaining its under-recognition among general surgical pathologists in routine practice. Encountering several cases that have illustrated the difficulty and confusion related to this finding, we decided to review our files for non-epithelial pulmonary lesions, both primary and metastatic, to critically address and illustrate this histological finding and discuss the sources of pitfall in the context of the differential diagnoses of biphasic pulmonary lesions.

## Material and methods

We searched our routine and consultation files for surgically excised lung metastases from malignant neoplasms other than carcinomas (including sarcoma of different types, embryonal tumors, and germ cell tumors) diagnosed in our institution between 2012 and 2018. Furthermore, our archive was searched for lung lesions diagnosed as adenofibroma, perivascular epithelioid cell tumor (PEComa), and solitary fibrous tumors (SFT). Diagnosis, site of origin, patient’s age, gender, and history of malignancy were drawn from the original pathology records. To have a representative overview of this morphological phenomenon, only surgical specimens were analyzed. In each case, all resected lung metastases have been reviewed and the presence, pattern, and extent of entrapped lung epithelium have been recorded (described as diffuse resulting in a biphasic pattern throughout versus focal and peripheral). Those cases with diffuse entrapment of respiratory epithelium were then used for further analysis. In most cases, immunohistochemical staining (CK7, TTF1, and NapsinA, in addition to other markers based on the exact diagnosis in a given case) was available. The overall histological appearance of the lesion was then described as adenofibroma-like, adenomyoepithelioma-like, biphasic synovial sarcoma–like, and other non-descript biphasic patterns.

## Results

### General clinical and demographic features

Forty-seven patients were retrieved for histological analysis. These comprised 38 patients with pulmonary metastases (81%) and 8 cases of primary pulmonary non-epithelial lesions (6 primary intrapulmonary SFTs, one SMARCB1-deficient fibromyxoid lung neoplasm of uncertain histogenesis, and one pulmonary adenofibroma). One PEComa could not be determined whether it is primary or metastatic due to lack of detailed clinical information. The 38 cases of lung metastases were from different sarcoma types (*n* = 35), germ cell tumors (*n* = 2), and Wilms tumor (*n* = 1). Age of the patients ranged between 2 and 84 years (median 52 years, mean 47 years). Twenty-one patients were females (44.7%). Eighteen patients suffered from ≥ 2 lung metastases; one patient had > 30 small lung metastases that were resected. Patient characteristics are shown in Table 1.

**Table 1:** Patient characteristics of the study cohort (47 patients)

No	Age years	Entrapment of alveolar respiratory epithelium	Histological pattern	Metastasis (number) vs primary	Site within the lung	Histological diagnosis	History of malignancy
1	33	No	No	MTS	Both sites	High-grade sarcoma NOS	High malignant epithelioid sarcoma NOS DDx Ewing sarcoma
2	17	No	No	MTS	Left superior lobe	Angiectatic osteosarcoma	Osteosarcoma right distal femur2014
3	66	No	No	MTS (M)	Right lobes, left inferior lobe	Leiomyosarcoma	Leiomyosarcoma
4	18	No	No	MTS (M)	Both sites	Osteosarcoma	Osteosarcoma 03/2016, resection of lung metastases 03/2017 and 10/2017
5	51	Diffuse	Fibroadenoma-like	MTS	Right and left inferior lobe	Leiomyosarcoma	Leiomyosarcoma of the uterus, bone metastasis 01/2017
6	63	No	No	MTS (2)	Left superior and inferior lobe	Synovial sarcoma	Synovial sarcoma left popliteal cavity 09/2015
7	72	Diffuse	Fibroadenoma-like	MTS (M)	Left superior and inferior lobe	Myxofibrosarcoma	Myxofibrosarcoma with lung metastases 10/2016, resection after radiochemotherapy
8	15	No	No	MTS	NOS	Osteosarcoma	Osteosarcoma distal tibia 11/2016
9	75	No	No	MTS	Right inferior lobe	Pleomorphic sarcoma	Recurrent UPS right shoulder 11/2015, synchronous carcinoid of the right lung, pericardial metastasis of the sarcoma
10	11	No	No	MTS	NOS	Ewing sarcoma/PNET family	Malignancy of Ewing sarcoma/PNET family 06/2015
11	72	No	No	MTS	Left inferior lobe	Synovial sarcoma	Synovial sarcoma right lower leg 2014
12	84	Peripheral	No	MTS	Right superior lobe	Leiomyosarcoma	Leiomyosarcoma of the mandible (lung metastases 2014 and 2016 resected), lung metastasized colorectal carcinoma 2014 (status post radiochemotherapy), prostate cancer
13	57	Peripheral	No	MTS (M)	NOS	Sarcoma NOS	Breast cancer 2015
14	11	No	No	MTS (M)	Both sites	Synovial sarcoma	Synovial sarcoma with multiple lung metastases (resected 2014, 2016 and 2017)
15	42	Diffuse	Adenomyoepithelioma-like	MTS (M, >30)	Right	Sarcomatous transformed atypical fibrous histiocytoma	Sarcomatous transformed atypical fibrous histiocytoma
16	43	No	No	MTS (M)	Right	Leiomyosarcoma	Leiomyosarcoma of the right parotid
17	50	No	No	MTS	Left superior lobe	High-grade sarcoma NOS	1999 leiomyosarcoma of the vagina
18	64	Peripheral	No	MTS	NOS	High-grade leiomyosarcoma	Status post tumor of the testis
19	58	No	No	MTS (M)	Right	Osteosarcoma	Undifferentiated pleomorphic sarcoma of left thigh 2013
20	66	No	No	MTS	NOS	UPS/myxoid fibrosarcoma grade 3	Myxoid fibrosarcoma grade 3 of the thigh 2009
21	14	Peripheral	No	MTS	Both sites	Low-grade fibromyxoid sarcoma	Low grade fibromyxoid sarcoma 2008, lung metastases resected 2014
22	35	Diffuse	Biphasic synovial sarcoma–like	MTS (M)	Both sites	Synovial sarcoma	Initial diagnosis of malignant peripheral nerve sheath tumor right foot (retrospectively monophasic synovial sarcoma), status post radiochemotherapy and amputation of the lower leg
23	52	No	No	MTS	Left superior lobe	Low-grade fibromyxoid sarcoma/ epithelioid fibrosarcoma	Low-grade fibromyxoid sarcoma/ epithelioid fibrosarcoma of the thigh
24	56	No	No	MTS (2)	Right superior and inferior lobe	Osteosarcoma	Osteosarcoma
25	60	No	No	MTS (M)	Left superior and inferior lobe	Leiomyosarcoma	High grade pleomorphic leiomyosarcoma (non-genital type) pelvic/retroperitoneal 2013, status post radiochemotherapy, resection of primary and of liver metastasis 2014
26	20	No	No	MTS	Right inferior lobe	Ewing sarcoma	Ewing sarcoma of the left thoracic wall
27	56	Diffuse	Fibroadenoma-like	MTS (M)	Right middle and inferior lobe	Sclerosing fibroblastic sarcoma	Sclerosing fibroblastic sarcoma retroperitoneal/pelvic wall, resection of lung metastases 2012 and 2013
28	70	Peripheral	No	MTS	Right inferior lobe	High-grade leiomyosarcoma	Leiomyosarcoma of the uterus 1991
29	48	No	No	MTS	Right superior lobe	Sarcoma NOS	Retroperitoneal SFT 2006
30	62	Diffuse	Fibroadenoma-like	Primary	NOS	Primary myxoid (myoepithelial!) neoplasm NOS (SMARCB1 deficiency)	Unknown
31	57	Peripheral	No	primary	Left inferior lobe	SFT	Unknown
32	66	Diffuse	Fibroadenoma-like	Primary	Pleura/lung	Intrapulmonary SFT (fibroadenoma-like variant)	Unknown
33	65	Peripheral	No	Primary	Pleura/lung	SFT	Malignant melanoma 2014, malignant biphasic mesothelioma (high grade)
34	20	Diffuse	Pulmonary blastoma–like	MTS	NOS	Embryonal rhabdomyosarcoma	Primary diagnosed in 2015, metastasis in 2017
35	64	Diffuse	Fibroadenoma-like	Primary	Right inferior lobe	Pulmonary adenofibroma	Unknown
36	2	Diffuse	Fibroadenoma-like	MTS (M)	Right superior lobe, right inferior lobe, left superior lobe	Wilms tumor	Wilms tumor at age of 2 years (10/2017), stage IV (lung metastases), status post neoadjuvant chemotherapy
37	54	No	No	MTS	Right middle lobe	Malignant bone tumor DDx osteosarcoma	1985 diagnosis of malignant “osteoblastoma” (radius), status post extirpation, re-extirpation due to recurrence, radiochemotherapy
38	66	Diffuse	Fibroadenoma-like	Uncertain	Left inferior lobe	Sclerosing cystic PEComa DD lung clear cell “sugar” tumor DDx metastasis of genital PEComa	Unknown
39	26	Diffuse	Fibroadenoma-like	MTS (2)	Right and left lung	Testicular teratoma	Concurrent testicular teratoma
40	31	No	No	MTS	Left inferior lobe	Germ cell tumor (embryonal carcinoma component)	Mixed germ cell tumor of the testis (embryonal carcinoma and seminoma and mature teratoma) with lung metastasis 2018
41	38	No	No	MTS	NOS	Atypical fibrous histiocytoma	2010 histiocytoma, 2014 recurrence, 2016 lung metastases
42	28	Diffuse	Fibroadenoma-like	MTS (M)	NOS	Sclerosing epithelioid fibrosarcoma of kidney	Unclassified tumor kidney region, treated as Wilms tumor
43	68	Diffuse	Fibroadenoma-like	Primary	Intrathorakal/lung	Partly intrapulmonary SFT (fibroadenoma like variant)	Unknown
44	48	Diffuse	Biphasic synovial sarcoma–like	MTS	NOS	Spindle cell sarcoma unclassified (adult fibrosarcoma)	Tumor of the lower extremity
45	41	No	No	MTS	NOS	MPNST	Recurrent MPNST peroneal nerve
46	62	No	No	Pleuropulmonary SFT	Lung/pleura (right lower lobe)	Recurrence of SFT with malignant (fibrosarcoma like) features	SFT (2012)
47	48	Peripheral	No	Primary	Right lower lobe	Intrapulmonary SFT, marked adenofibroma like features	Unknown

*DDx*, differential diagnosis; *MPNST*, malignant peripheral nerve sheath tumor; *MTS*, metastasis; (*M*), multiple; *NA*, not assessable; *NOS*, not otherwise specified; *PEComa*, perivascular epithelioid cell tumor; *PNET*, primitive neuroectodermal tumor; *SFT*, solitary fibrous tumor; *UPS*, undifferentiated pleomorphic sarcoma

### Frequency of respiratory/alveolar epithelium entrapment

**Fig. 1:**
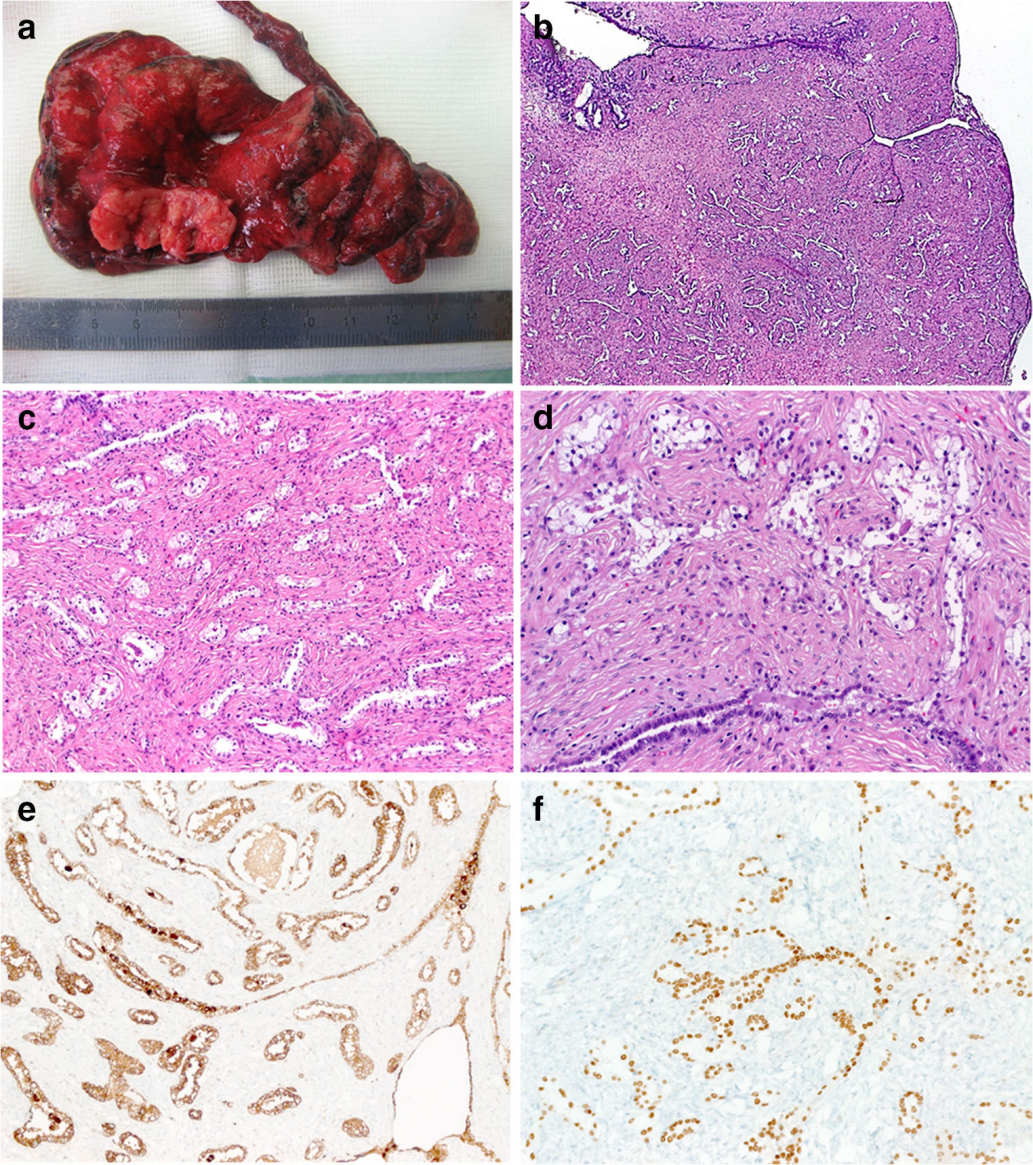
**a** The single genuine case of pulmonary adenofibroma presented as well circumscribed whitish nodule (lower field). Histology showed biphasic fibroadenoma-like pattern (**b**). At high power, branching tubules lined by clear epithelial cells (**c**) with adjacent spindle cell stroma (**d**). The epithelial component expresses NapsinA (**e**) and TTF1 (**f**)

**Fig. 2:**
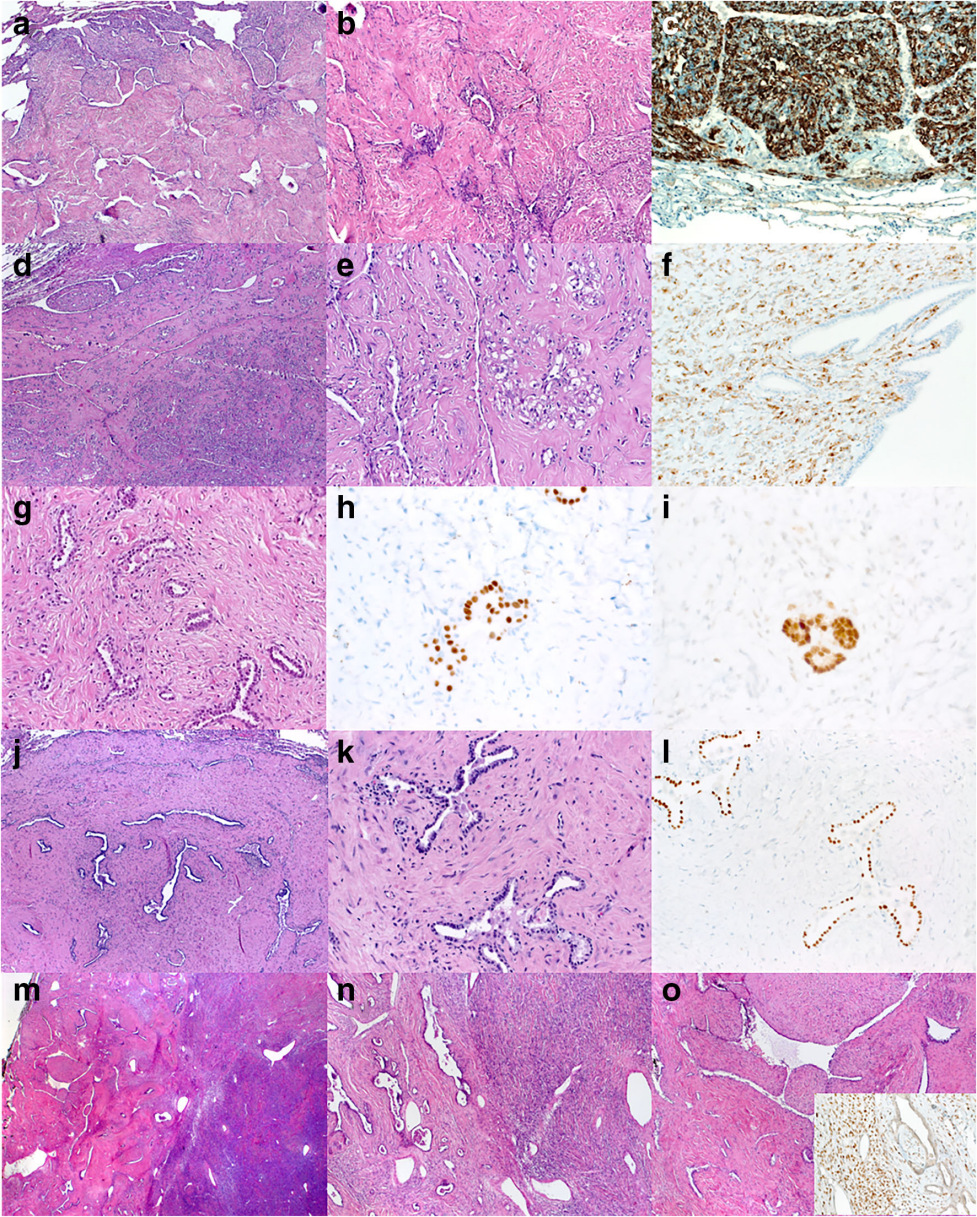
Examples of primary and metastatic lung lesions closely mimicking (almost indistinguishable from) pulmonary adenofibroma. **a**–**c** Lung metastasis of sclerosing epithelioid fibrosarcoma (**a** and **b**: H&E; **c**: MUC4). **d**–**f** Intrapulmonary PEComa (**d** and **e**: H&E; **f**: HMB45). **g** Metastatic Wilms tumor containing both TTF1+/PAX8- entrapped respiratory epithelium (**h**) and PAX8+/TTF1- neoplastic epithelium (**i**). **j**–**l** Two adenofibroma-like lesions (**j**, **k**) were detected at same time as a histologically identical mature testicular teratoma (**j**, **k**: H&E; **l**: TTF1). Examples of cellular (**m**) and sclerosing (**n**) solitary fibrous tumor, both had prominent adenofibroma-like areas (seen on the left in both images). **o** Higher magnification of the adenofibroma-like area. Strong expression of STAT6 in the stromal cells is seen in **o** (inset)

**Fig. 3:**
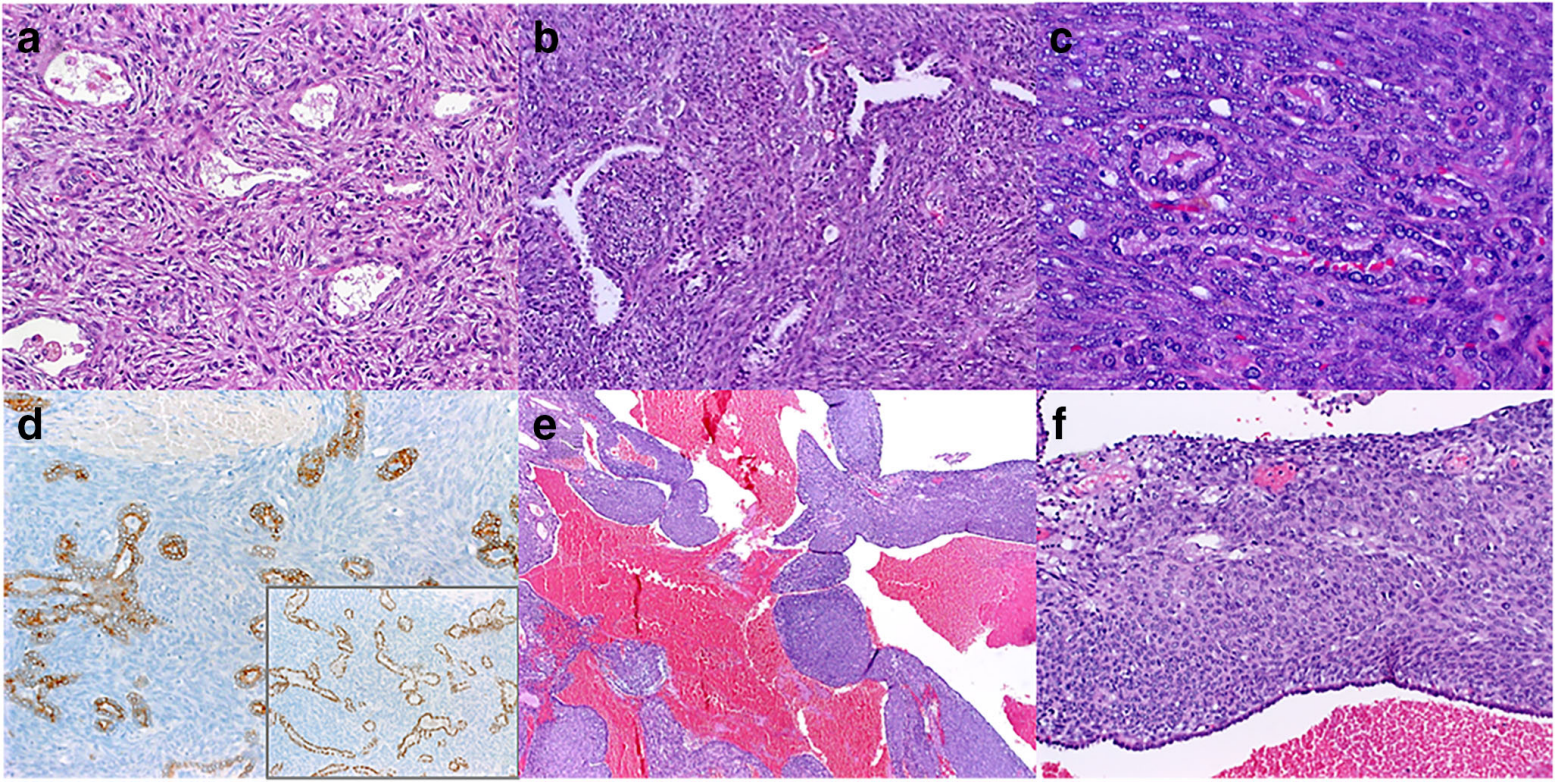
Examples of metastatic mesenchymal neoplasms/sarcomas with prominent epithelial entrapment mimicking biphasic primary pulmonary neoplasms. Metastatic atypical fibrous histiocytoma with features mimicking adenomyoepithelioma (**a**). Metastatic unclassified spindle cell sarcoma (**b**) and monophasic spindle cell synovial sarcoma (**c**) mimicking biphasic synovial sarcoma. Expression of CK7 (**d**) and TTF1 (**d** inset) highlighting entrapped epithelium and confirming its pulmonary origin. **e**, **f** Lung metastasis of embryonal rhabdomyosarcoma displaying solid and cystic pattern closely mimicking type 2 pleuropulmonary blastoma

In total, 23 out of 47 cases (49%) showed entrapment of respiratory/alveolar epithelium with variable extent. In eight of these cases, entrapped pulmonary epithelium was seen mainly in the peripheral portion of the tumor and was clearly evident as secondary entrapment of lung tissue as the tumor grows peripherally. The remainder (*n* = 15), which represent the basis of the subsequent morphological analysis, showed prominent entrapped alveolar and respiratory epithelium that was seen throughout the lesion albeit to varying extent. These entrapped glands frequently showed reactive/regenerative appearance with occasional hobnail-like nuclear prominence, vesicular chromatin, and irregular configuration mimicking neoplastic glands, but lacked significant cytological atypia. The gland size varied greatly from small acinar-type glands or microcystic spaces lined by flattened epithelial cells and containing mucoid secretion to branching leaflet-like papillary spaces. The overall appearance of these glands was different from normal alveoli, thus enhancing their confusion with true neoplastic glands. Based on the cellularity of the background neoplasm, four different histological patterns were recognized: (1) lesions closely mimicking PAF (11/15); (2) those closely mimicking adenomyoepithelioma/epithelial-myoepithelial carcinoma (1/15); (3) biphasic synovial sarcoma–like (2/15); and (4) pulmonary blastoma–like (1/15) patterns (Figs. 1, 2, and 3, Table 2).

**Table 2:** Entrapment of alveolar/respiratory epithelium in different primary and metastatic pulmonary lesions

Category	Subcategory	No of cases	Entrapment of lung epithelium	No of cases	Number of adenofibroma-like cases	Number of adenomyoepithelial-like cases	Number of biphasic synovial sarcoma–like cases	Number of pulmonary blastoma–like cases
Lung primary		8	Peripheral	3	0	0	0	0
			Diffuse	4	4	0	0	0
	SFT	6	Peripheral	3	0	0	0	0
			Diffuse	2	2	0	0	0
	Pulmonary adenofibroma	1	Diffuse	1	1	0	0	0
	Myxoid sarcomatoid neoplasm NOS of the lung (SMARCB1 deficient)	1	Diffuse	1	1	0	0	0
Primary or metastasis	Sclerosing cystic PEComa	1	Diffuse	1	1	0	0	0
Lung metastasis		38	Peripheral	5	0	0	0	0
			Diffuse	10	6	1	2	1
	Leiomyosarcoma	7	Peripheral	3	0	0	0	0
			Diffuse	1	1	0	0	0
	Ewing sarcoma/PNET family	2	Peripheral	0	0	0	0	0
			Diffuse	0	0	0	0	0
	Osteosarcoma/malignant bone tumor	6	Peripheral	0	0	0	0	0
			Diffuse	0	0	0	0	0
	Synovial sarcoma	4	Peripheral	0	0	0	0	0
			Diffuse	1	0	0	1	0
	Myxofibrosarcoma	1	Diffuse	1	1	0	0	0
	Pleomorphic sarcoma/myxoid fibrosarcoma grade 3	2	Peripheral	0	0	0	0	0
			Diffuse	0	0	0	0	0
	Embryonal rhabdomyosarcoma	1	Diffuse	1	0	0	0	1
	Low-grade fibromyxoid sarcoma	2	Peripheral	1	0	0	0	0
			Diffuse	0	0	0	0	0
	Sarcoma NOS	5	Peripheral	1	0	0	0	0
			Diffuse	1	1	0	0	0
	Atypical fibrous histiocytoma	2	Peripheral	0	0	0	0	0
			Diffuse	1	0	1	0	0
	Sclerosing epithelioid fibrosarcoma	1	Diffuse	1	1	0	0	0
	Wilms tumor	1	Diffuse	1	1	0	0	0
	Germ cell tumor	2	Peripheral	0	0	0	0	0
			Diffuse	1	1	0	0	0
	Spindle cell sarcoma unclassified (adult fibrosarcoma)	1	Diffuse	1	0	0	1	0
	MPNST	1	Peripheral	0	0	0	0	0
			Diffuse	0	0	0	0	0

*MPNST*, malignant peripheral nerve sheath tumor; *NOS*, not otherwise specified; *PEComa*, perivascular epithelioid cell tumor; *PNET*, primitive neuroectodermal tumor; *SFT*, solitary fibrous tumor

#### Adenofibroma-like pattern

This pattern (seen in 11/15 cases; 73%) was characterized by the presence of a diffuse component of branching leaflet-like variably dilated glands throughout the lesion imparting a characteristic adenofibroma-like, phylloides-like or fibroepithelial hamartoma–like pattern. This feature (which is restricted to spindle cell neoplasms with low-grade histological features and paucicellular sclerosing stroma) was seen in 4 primary intrapulmonary lesions (2 SFTs, 1 unclassified myxoid lesion, and 1 pulmonary adenofibroma), 1 PEComa (not clear if primary or metastatic), 4 metastases from different sarcoma types including multiple metastases from sclerosing epithelioid fibrosarcoma, 1 metastatic Wilms tumor, and 1 patient with two PAF-like lung nodules concurrent to a histologically identical mature testicular teratoma (Table 2). Notably, the Wilms tumor metastasis (post-treatment) contained prominent fibrous stroma entrapping an admixture of native respiratory epithelium as well as minute glands, positive for PAX8, negative with TTF1, indicating neoplastic epithelial origin. The metastatic sarcoma category contained different entities, but (except 1 leiomyosarcoma) all were of presumable fibroblastic histogenesis. Representative examples of these entities are illustrated in Fig. 2.

The two lung nodules from the patient with concurrent mature testicular teratoma showed concordant histology between the mesenchymal stromal component of both the testicular lesion and the lung nodules; both expressed desmin, smooth muscle actin (SMA), and pancytokeratin, but the epithelial components were discordant (Fig. 2j–l). Although it is impossible to definitely classify the lung lesions of this patient without molecular testing for chromosome 12p amplification, the concordant phenotypes and the clinical presentation are more consistent with metastatic disease but this remains unsolved.

Among the PAF-like lesions, only a single case in this group qualified as genuine PAF (1/11; 9%). The spindled stroma of this case expressed SMA while the entrapped epithelium was positive for NapsinA and TTF1 (Fig. 1). Both of epithelial and stromal components were negative with estrogen and progesterone receptors, STAT6, and marker for adenocarcinoma of the lung (MAdL).

#### Adenomyoepithelial-like pattern

One case of metastatic atypical fibrous histiocytoma presented as a moderately cellular spindle cell lesion entrapping numerous small acinar respiratory glands mimicking adenomyoepithelioma (Fig. 3a). Diagnosis of metastatic atypical fibrous histiocytoma would have been impossible without knowing the clinical history.

#### Biphasic synovial sarcoma–like pattern

A highly cellular spindle cell sarcoma with prominent evenly distributed small glands mimicking biphasic synovial sarcoma was seen in two examples: one patient with multiple lung metastases of monophasic spindle cell synovial sarcoma (Fig. 3c) and another with lung metastasis from unclassified fibrosarcoma-like spindle cell sarcoma (Fig. 3b). Positive CK7 and TTF1 stains ruled out neoplastic epithelial elements in both cases (Fig. 3d).

#### Pulmonary blastoma–like pattern

This least common pattern was seen in a case of lung metastasis from embryonal rhabdomyosarcoma that contained prominent entrapped cystic spaces lined by respiratory epithelium closely mimicking type II pleuropulmonary blastoma (Fig. 3e, f). Notably, this case was subjected to NGS testing and lacked *DICER1* mutations, thus arguing against the possibility of primary metachronous pleuropulmonary blastoma in the setting of DICER1 syndrome.

## Discussion

It is well known that cancer metastasis may reveal diverse secondary morphological patterns that contribute to the differential diagnostic confusion in a given case. This is particularly true for lung metastasis where metastatic deposits may entrap native respiratory epithelium closely mimicking a biphasic neoplasm. The confusion is further enhanced by the frequent observation of prominent reactive and/ or regenerative changes of entrapped native epithelial glands closely mimicking neoplastic elements. Over the years, we have encountered several metastatic lung lesions that have been mistaken for primary pulmonary malignancies (carcinosarcoma, adenomyoepithelial carcinoma, etc.) based on prominent entrapped native glandular component where the alveolar epithelial immunophenotype was misinterpreted as evidence of pulmonary origin. This prompted us to perform the current study.

In the present study, we identified and illustrated different morphological patterns adopted by primary or metastatic non-epithelial neoplasms in the lung. Based on the degree of cellularity and other characteristics of the neoplastic mesenchymal component and the pattern of entrapped native respiratory epithelium, the tumors we have analyzed closely mimicked a variety of benign or malignant, primary or metastatic pulmonary neoplasms. Exploration of the previous clinical history/imaging combined with careful assessment of the stromal characteristics for phenotypic hints was the key to correct diagnosis.

The biphasic pattern in lung metastasis, particularly from low-grade non-epithelial neoplasms/sarcomas, frequently obscures the original morphological pattern seen in the primary tumor and, instead, closely mimics benign or harmless hamartomatous lesions. *Pulmonary adenofibroma* (PAF) is the most frequent and the most striking and misleading pattern encountered in this study. Paucicellular intrapulmonary SFT is the main representative in this category. While some SFTs contain only focal PAF-like areas and are thus easily recognizable as adenofibromatous SFT variants, others were uniformly PAF-like. Their immunoprofile is otherwise indistinguishable from conventional SFTs. PAF-like SFTs represented up to 24% of intrapulmonary SFTs in a previous series [6]. Following discovery of *STAT6-NAB2* gene fusions as driver events in most of SFTs, STAT6 IHC has emerged as highly sensitive and specific marker for SFT [7–9]. In the pre-STAT6 era however, many PAF-like SFTs were misclassified as PAF. This is because PAFs and SFTs otherwise share expression of CD99, CD34, bcl-2, and vimentin in their mesenchymal component [5, 10, 11]. Notably, 71% of PAFs were STAT6-positive and showed *NAB2-STAT6* rearrangement, confirming PAF-like SFT [5]. In a previous study, 71% of PAFs expressed hormone receptors in the stroma but all SFTs were negative for estrogen receptor-α by IHC [5]. Based on these recent observations and our current study, the concept of PAF as a specific entity is questionable as most of putative PAFs seem to be classifiable as other distinctive entities. The molecular pathogenesis and histogenesis of the vanishingly rare genuine “PAF lesions” remains to be further studied.

Other entities that closely mimicked PAF in this study include metastatic Wilms tumor, mature teratoma, and sclerosing epithelioid fibrosarcoma. Admittedly, many of these entities would have been impossible to diagnose by morphology alone if the clinical history was not available and/or STAT6 (in cases of intrapulmonary SFT) IHC was not applied.

In this context, it is worth mentioning that lung metastasis from biphasic neoplasms (Müllerian adenosarcomas and malignant fibroepithelial tumors of breast, prostate, etc.) usually contains only the mesenchymal stromal component. Biphasic synovial sarcoma may be the main exception to this. Accordingly, any glandular component in a biphasic lung lesion should be considered to represent entrapped native epithelium until proven otherwise. Immunophenotyping of the epithelial glandular component and careful assessment of atypia within the glands should allow distinguishing reactive entrapped glands from genuine neoplastic epithelial component.

On the other hand, SFTs with high cellularity and spindle cell sarcomas metastatic to the lung might be mistaken for biphasic synovial sarcoma. In contrast to biphasic synovial sarcoma, the entrapped alveolar glands show consistent pneumocytic phenotype, which is not the case in synovial sarcoma glands (the latter are CK7+, TLE1+, TTF1-, NapsinA-). Furthermore, the presence of alveolar-type glands with prominent regenerative atypia can closely mimic primary biphasic lung malignancies, in particular carcinosarcoma, primary and metastatic sarcomatoid (dedifferentiated) adenocarcinoma, and adenomyoepithelioma (epithelial-myoepithelial carcinoma). Carcinosarcoma (sarcomatoid carcinoma) typically features frankly malignant glands and high-grade cytology in both components, and the stromal component is usually highly pleomorphic. Pulmonary adenomyoepithelioma may represent primary lung neoplasm or metastasis from a salivary gland primary (epithelial-myoepithelial carcinoma) [12]. Recognition of the myoepithelial phenotype in the stromal component is helpful in diagnosis as well as the clinical history. In addition, the spatial arrangements of the epithelium surrounded by clear cell myoepithelial component are typical. In doubtful cases, it seems that *HRAS* mutation testing is context-specific for epithelial-myoepithelial carcinoma [12]. The rare pneumocytic adenomyoepithelioma, which contains similar alveolar-type neoplastic glands associated with myoepithelial stromal component, might be challenging. However, the neoplastic nature of the epithelial component in pneumocytic adenomyoepithelioma has been recently questioned [13].

In summary, we herein illustrated pitfalls related to frequent florid entrapment of native pulmonary epithelium within primary and metastatic non-epithelial neoplasms closely mimicking a biphasic lesion and occasionally leading to erroneous diagnosis of a primary pulmonary neoplasm based on the pneumocytic immunophenotype of the glandular component. As genuine PAF is exceptionally rare and a PAF-like pattern can be frequently seen in a variety of primary (benign or malignant) and metastatic lung tumors, any PAF-like lung lesion should be approached very critically and the recent and remote clinical history evaluated for any extrapulmonary neoplasm. Accordingly, PAF should be considered *a diagnosis by exclusion*. Misdiagnosis of metastatic malignancies as a “harmless PAF” would have significant prognostic and therapeutic implications.
